# Why the Gap Persists: A Systematic Review of Sociocultural, Clinical, and Systemic Factors Driving Maternal Health Disparities Between Black and White Women in the United Kingdom

**DOI:** 10.7759/cureus.114480

**Published:** 2026-08-13

**Authors:** Sunday C Adeyemo, Eniola D Olabode, Kehinde Awodele, Adeola D Aderinwale, Ayodele Ajayi, Christianah A Olaniran, Olusayo E Oniwinde

**Affiliations:** 1 Health and Social Care, QA Higher Education, London, GBR; 2 Public Health Research, University of Wolverhampton, Wolverhampton, GBR; 3 Health and Biomedical Sciences, The Higher Institute of Health (Institut Supérieur de Santé Niger), Niamey, NER; 4 Community Medicine, The Higher Institute of Health (Institut Supérieur de Santé Niger), Niamey, NGA; 5 Obstetrics and Gynaecology, Osun State University, Osogbo, NGA; 6 Public Health, Osun Primary Health Care Development Board, Osogbo, NGA; 7 Nursing, Pear Tree Court Care Home, Waterlooville, GBR; 8 Psychiatry, Afe Babalola University Multi-System Hospital, Ado-Ekiti, NGA; 9 Nursing, Osun State University, Osogbo, NGA; 10 Health, Society and Well-being, University of Wolverhampton, Wolverhampton, GBR

**Keywords:** black women, clinical factors, ethnic inequalities, maternal health disparities, sociocultural factors, systematic review, systemic factors, uk maternity care

## Abstract

Despite overall low maternal mortality rates in the United Kingdom, Black women are approximately four times more likely to die during pregnancy, childbirth, or the postnatal period than White women. This disparity has persisted for over a decade despite national initiatives. This systematic review aimed to synthesize evidence from 2010-2025 on sociocultural, clinical, and systemic factors contributing to maternal health disparities between Black and White women in the United Kingdom (UK). Databases such as MEDLINE, Embase, Cumulative Index to Nursing and Allied Health Literature (CINAHL), PsycINFO, and Web of Science (January 2010-December 2025) were searched along with grey literature sources including EthOS, OpenGrey, Mothers and Babies: Reducing Risk through Audits and Confidential Enquiries across the UK (MBRRACE-UK), Birthrights, and NHS Race and Health Observatory. We included primary research studies (quantitative, qualitative, or mixed-methods) published in English that examined maternal health outcomes, experiences, or carer processes for Black and White women in the UK and analyzed at least one sociocultural, clinical, or systemic factor associated with disparities. Two reviewers independently screened titles/abstracts and full texts in Rayyan, extracted data using a piloted form, and assessed quality using Joanna Briggs Institute (JBI) checklists, Critical Appraisal Skills Programme (CASP), and Mixed Methods Appraisal Tool (MMAT). Narrative synthesis was conducted. Thirty-six studies were included, with 66.7% published post-2020. Sociocultural factors centered on experiences of discrimination and "not being listened to," which eroded trust and delayed care-seeking. Clinical factors confirmed higher prevalence of hypertensive disorders, pre-eclampsia, gestational diabetes, and postpartum hemorrhage in Black women; however, disparities in escalation and response persisted after adjusting for comorbidities. Systemic issues included inconsistent continuity of care implementation, workforce underrepresentation, and a "postcode lottery" in service provision. The evidence demonstrates that disparities arise from the interaction of all three domains rather than any single factor. Multi-level interventions addressing sociocultural, clinical, and systemic factors simultaneously are essential. Future priorities include continuity of carer models, discrimination-focused longitudinal studies, and implementation science research. The persistence of these disparities requires sustained commitment from clinicians, policymakers, and researchers to translate evidence into meaningful action.

## Introduction and background

The maternal mortality ratio is low in the United Kingdom (UK), but there are significant ethnic differences. Black women are around four times more likely to die during pregnancy, childbirth, and the postnatal period than White women, with a maternal mortality rate of 35.9 per 100,000 maternities compared to 8.9 per 100,000 for White women [1]. Although various national initiatives and policy pledges have been made to correct the disparity, it has not been statistically different for more than 10 years [1]. Maternity care in the UK is delivered through the National Health Service (NHS), which provides free care at the point of access to all residents. This universal healthcare model makes the persistent ethnic disparities in outcomes particularly striking, as financial barriers to care are largely eliminated, pointing to structural and systemic issues within the care delivery system itself. The 2021-2023 Mothers and Babies: Reducing Risk through Audits and Confidential Enquiries across the UK (MBRRACE-UK) report, which is the national surveillance programme responsible for monitoring maternal deaths in the UK, showed Black women had a more than twofold greater risk of death than White women [2].

Ethnic inequalities in maternal, neonatal and infant health care are systematic differences in health outcomes and experiences between ethnic/racial groups that are unjust and preventable, grounded in social and structural determinants of health rather than genetic or intrinsic biological differences [3]. The inequalities are well documented in the UK with only slight improvements since the first Confidential Enquiry into Maternal and Child Health was published in 2007 [4]. These sustained disparities are not due to any single clinical risk factor but are multifaceted. Black women registered in the UK have the highest incidence of comorbidities including hypertension and pre-eclampsia, but these disparities are not the whole story behind the inequities in mortality and severe morbidity [5]. There is growing evidence that three domains of interaction exist: sociocultural, clinical and systemic [5-7].

Sociocultural factors are experiences of discrimination, lack of being heard, dismissive pain reaction, and distrust of health care. The Birthrights investigation 'Systemic racism, not broken bodies' found that Black women's concerns were not being taken seriously and symptoms were not being acted upon swiftly enough during pregnancy and birth [6]. These experiences shape care-seeking behaviour such as postponing early booking for antenatal care and low uptake of services. MacLellan et al. [7] conducted a qualitative evidence synthesis that revealed that discriminatory practices and communication failures in NHS maternity services in the UK are causing problems for ethnic minority women. Clinical factors include differences in disease prevalence and differences in the delivery of care. Data from the UK show a higher prevalence of hypertensive disorders, gestational diabetes, and postpartum hemorrhage in Black women [5]. There are also indications that clinical pathways do not work equally among ethnic groups regarding timely diagnosis, escalation of care, and management of obstetric emergencies [8-11]. Systemic factors are associated with the organization and provision of maternity services. These encompass underrepresentation of Black and minority ethnic staff in senior clinical and leadership positions, not having an effective continuity of carer model [12-15], inconsistent access to translation services [16], and a 'postcode lottery' in service provision [7,17,18]. While the NHS Long Term Plan and Equity and Equality guidance have made continuity of carer a priority for Black and Asian women, this has yet to be fully achieved [13,19].

Although there is increasing evidence and policy interest in why the mortality and morbidity gap persists, existing evidence remains fragmented across the three domains. Individual studies have examined sociocultural, clinical, or systemic factors separately, but no systematic review has yet synthesized evidence across all three domains to provide a comprehensive understanding of the maternal health disparity gap for Black women in the UK. A comprehensive synthesis is needed to support the development of effective, multi-level interventions that address the interconnected nature of these factors. Given the anticipated heterogeneity in study designs (including quantitative, qualitative, and mixed-methods studies), outcome measures, and analytical approaches across the included studies, a meta-analysis was not feasible. A narrative synthesis approach was therefore selected as it allows for the integration of diverse evidence types and facilitates exploration of relationships within and across the three domains of interest [20].

Thus, this systematic review aimed to identify, appraise, and synthesize evidence from January 1, 2010 to December 31, 2025 reporting sociocultural, clinical, and systemic factors that may explain disparities in maternal outcomes between Black and White women in the UK. This systematic synthesis aims to identify the evidence that is currently available, identify gaps in the evidence base, and examine how these domains interconnect to reinforce inequities.

## Review

Materials and methods

Eligibility Criteria

Studies were considered for inclusion if they met the following criteria: they were peer-reviewed primary research articles published in English between January 1, 2010 and December 31, 2025; reported on maternal health outcomes, experiences, or care processes for Black and White women in the UK; examined at least one sociocultural, clinical, or systemic factor associated with disparities; and used quantitative, qualitative, or mixed-methods designs.

Studies were excluded if they did not provide disaggregated data by ethnicity, focused solely on neonatal outcomes without maternal data, were conducted outside the UK, were published outside the specified date range, or were not published in English.

Search Strategy

Databases including MEDLINE, Embase, Cumulative Index to Nursing and Allied Health Literature (CINAHL), PsycINFO and Web of Science were searched from January 1, 2010 to December 31, 2025. EthOS and OpenGrey, and websites of key UK maternal health organisations such as MBRRACE-UK, Birthrights and NHS Race and Health Observatory were used to identify grey literature. The full search string is presented in Table 1.

**Table 1:** Database search strings This table details the complete search strategies used for each electronic database in this systematic review. Searches combined controlled vocabulary (where available) and free-text terms across four key concepts: ethnicity/population, maternal health, geographic location (UK), and factors (sociocultural, clinical, and systemic). Database-specific syntax, field tags, and proximity operators were employed as indicated. All searches were restricted to English language publications from January 1, 2010, to December 31, 2025. The right-hand column lists additional platform-specific filters applied during the search process.

Database (Platform)	Search String	Filters/Limits
PubMed (MEDLINE)	("Ethnicity"[Mesh] OR "Ethnic and Racial Minorities"[Mesh] OR "Minority Groups"[Mesh] OR "Black People"[Mesh] OR "Racial Groups"[Mesh] OR "Healthcare Disparities"[Mesh] OR "Health Status Disparities"[Mesh] OR "racial disparit*"[tiab] OR "ethnic minorit*"[tiab] OR "BAME"[tiab] OR "Black women"[tiab] OR "minoritised"[tiab] OR "minoritized"[tiab]) AND ("Maternal Health"[Mesh] OR "Maternal Health Services"[Mesh] OR "Maternal Mortality"[Mesh] OR "Pregnancy Outcome"[Mesh] OR "Pregnancy"[Mesh] OR "Perinatal Care"[Mesh] OR "Prenatal Care"[Mesh] OR "Postpartum Period"[Mesh] OR "maternal health"[tiab] OR "maternity care"[tiab] OR "pregnancy outcome*"[tiab] OR "perinatal"[tiab] OR "antenatal"[tiab] OR "postnatal"[tiab] OR "postpartum"[tiab] OR "childbirth"[tiab] OR "obstetric*"[tiab]) AND ("United Kingdom"[Mesh] OR "England"[Mesh] OR "Scotland"[Mesh] OR "Wales"[Mesh] OR "Northern Ireland"[Mesh] OR "UK"[tiab] OR "Britain"[tiab] OR "British"[tiab]) AND ("sociocultural factor*"[tiab] OR "discrimination"[tiab] OR "racism"[tiab] OR "systemic racism"[tiab] OR "implicit bias"[tiab] OR "trust"[tiab] OR "continuity of care"[tiab] OR "continuity of carer"[tiab] OR "interpreter services"[tiab] OR "language barrier*"[tiab] OR "comorbidit*"[tiab] OR "hypertension"[tiab] OR "pre-eclampsia"[tiab] OR "postpartum hemorrhage"[tiab] OR "gestational diabetes"[tiab] OR "workforce diversity"[tiab] OR "postcode lottery"[tiab])	• Date: 2010-2025 • Language: English • Humans
Embase (Ovid)	(exp ethnic group/ OR exp ethnicity/ OR exp racial disparity/ OR exp minority group/ OR exp black person/ OR (ethnic minorit* or racial minorit* or BAME or Black or Asian or minority group* or racial disparit* or ethnic disparit*).ti,ab.) AND (exp maternal health/ OR exp maternity care/ OR exp prenatal care/ OR exp postnatal care/ OR exp perinatal care/ OR exp pregnancy complication/ OR exp obstetric care/ OR exp maternal mortality/ OR exp pregnancy outcome/ OR (maternal health or maternity care or pregnancy outcome* or perinatal care or antenatal care or postnatal care or postpartum or childbirth or obstetric*).ti,ab.) AND (exp United Kingdom/ OR exp England/ OR exp Scotland/ OR exp Wales/ OR exp Northern Ireland/ OR (UK or U.K. or Britain or British or England or Scotland or Wales or Northern Ireland).ti,ab.) AND (exp discrimination/ OR exp racism/ OR exp cultural competence/ OR exp language barrier/ OR exp health care access/ OR exp health inequality/ OR (sociocultural factor* or discrimination or racism or systemic racism or implicit bias or trust or continuity of care or continuity of carer or workforce diversity or interpreter services or language barrier* or comorbidit* or hypertension or pre-eclampsia or postpartum hemorrhage or gestational diabetes).ti,ab.)	• Date: 2010-2025 • Language: English • Humans • Exclude conference abstracts
CINAHL (EBSCOhost)	(MH "Ethnic Groups") OR (MH "Minority Groups") OR (MH "Black People") OR (MH "Racial Disparities") OR (MH "Health Status Disparities") OR TI (ethnic minorit* OR racial minorit* OR BAME OR Black OR Asian OR minority group* OR racial disparit* OR ethnic disparit*) OR AB (ethnic minorit* OR racial minorit* OR BAME OR Black OR Asian OR minority group* OR racial disparit* OR ethnic disparit*) AND (MH "Maternal Health") OR (MH "Maternal Health Services") OR (MH "Maternal Mortality") OR (MH "Perinatal Care") OR (MH "Prenatal Care") OR (MH "Postnatal Care") OR (MH "Pregnancy Outcomes") OR (MH "Pregnancy Complications") OR TI (maternal health OR maternity care OR pregnancy outcome* OR perinatal OR antenatal OR postnatal OR postpartum OR childbirth OR obstetric*) OR AB (maternal health OR maternity care OR pregnancy outcome* OR perinatal OR antenatal OR postnatal OR postpartum OR childbirth OR obstetric*) AND (MH "United Kingdom") OR (MH "England") OR (MH "Scotland") OR (MH "Wales") OR (MH "Northern Ireland") OR TI (UK OR U.K. OR Britain OR British OR England OR Scotland OR Wales OR Northern Ireland) OR AB (UK OR U.K. OR Britain OR British OR England OR Scotland OR Wales OR Northern Ireland) AND (MH "Socioeconomic Factors") OR (MH "Discrimination") OR (MH "Racism") OR (MH "Implicit Bias") OR (MH "Language Barriers") OR (MH "Continuity of Patient Care") OR TI (sociocultural factor* OR discrimination OR racism OR systemic racism OR implicit bias OR trust OR continuity of care OR continuity of carer OR interpreter services OR language barrier* OR comorbidit* OR hypertension OR pre-eclampsia OR postpartum hemorrhage OR gestational diabetes) OR AB (sociocultural factor* OR discrimination OR racism OR systemic racism OR implicit bias OR trust OR continuity of care OR continuity of carer OR interpreter services OR language barrier* OR comorbidit* OR hypertension OR pre-eclampsia OR postpartum hemorrhage OR gestational diabetes)	• Date: 2010-2025 • Language: English • Research Articles • Humans
PsycINFO (Ovid)	(exp ethnic groups/ OR exp minority groups/ OR exp racial and ethnic groups/ OR (ethnic minorit* or racial minorit* or BAME or Black or Asian or minority group* or racial disparit* or ethnic disparit*).ti,ab.) AND (exp maternal health/ OR exp maternity care/ OR exp prenatal care/ OR exp postnatal care/ OR exp perinatal care/ OR exp pregnancy/ OR exp childbirth/ OR (maternal health or maternity care or pregnancy outcome* or perinatal care or antenatal care or postnatal care or postpartum or childbirth or obstetric*).ti,ab.) AND (exp United Kingdom/ OR (UK or U.K. or Britain or British or England or Scotland or Wales or Northern Ireland).ti,ab.) AND (exp discrimination/ OR exp racial discrimination/ OR exp prejudice/ OR exp health care seeking behavior/ OR exp trust/ OR (sociocultural factor* or discrimination or racism or systemic racism or implicit bias or trust or continuity of care or continuity of carer or interpreter services or language barrier* or comorbidit* or hypertension or pre-eclampsia or postpartum hemorrhage or gestational diabetes).ti,ab.)	• Date: 2010-2025 • Language: English • Humans • Empirical Studies
Web of Science	(TS=("ethnicity" OR "ethnic minorit*" OR "racial minorit*" OR "minority groups" OR "black" OR "asian" OR "bame" OR "racial disparit*" OR "ethnic disparit*" OR "black women" OR "minoritised" OR "minoritized") AND TS=("maternal health" OR "maternity care" OR "pregnancy outcome*" OR "perinatal" OR "antenatal" OR "postnatal" OR "postpartum" OR "childbirth" OR "obstetric*" OR "maternal mortality" OR "maternal morbidity") AND TS=("UK" OR "United Kingdom" OR "Britain" OR "British" OR "England" OR "Scotland" OR "Wales" OR "Northern Ireland") AND TS=("sociocultural factor*" OR "discrimination" OR "racism" OR "systemic racism" OR "implicit bias" OR "trust" OR "continuity of care" OR "continuity of carer" OR "interpreter services" OR "language barrier*" OR "comorbidit*" OR "hypertension" OR "pre-eclampsia" OR "postpartum hemorrhage" OR "gestational diabetes" OR "workforce diversity" OR "postcode lottery" OR "service provision" OR "health inequalities"))	• Date: 2010-2025 • Language: English • Articles, Reviews • Research Areas: Obstetrics, Public Health, Nursing, Social Sciences
CINAHL (EBSCOhost)	(MH "Ethnic Groups") OR (MH "Minority Groups") OR (MH "Black People") OR (MH "Racial Disparities") OR (MH "Health Status Disparities") OR TI (ethnic minorit* OR racial minorit* OR BAME OR Black OR Asian OR minority group* OR racial disparit* OR ethnic disparit*) OR AB (ethnic minorit* OR racial minorit* OR BAME OR Black OR Asian OR minority group* OR racial disparit* OR ethnic disparit*) AND (MH "Maternal Health") OR (MH "Maternal Health Services") OR (MH "Maternal Mortality") OR (MH "Perinatal Care") OR (MH "Prenatal Care") OR (MH "Postnatal Care") OR (MH "Pregnancy Outcomes") OR (MH "Pregnancy Complications") OR TI (maternal health OR maternity care OR pregnancy outcome* OR perinatal OR antenatal OR postnatal OR postpartum OR childbirth OR obstetric*) OR AB (maternal health OR maternity care OR pregnancy outcome* OR perinatal OR antenatal OR postnatal OR postpartum OR childbirth OR obstetric*) AND (MH "United Kingdom") OR (MH "England") OR (MH "Scotland") OR (MH "Wales") OR (MH "Northern Ireland") OR TI (UK OR U.K. OR Britain OR British OR England OR Scotland OR Wales OR Northern Ireland) OR AB (UK OR U.K. OR Britain OR British OR England OR Scotland OR Wales OR Northern Ireland) AND (MH "Socioeconomic Factors") OR (MH "Discrimination") OR (MH "Racism") OR (MH "Implicit Bias") OR (MH "Language Barriers") OR (MH "Continuity of Patient Care") OR TI (sociocultural factor* OR discrimination OR racism OR systemic racism OR implicit bias OR trust OR continuity of care OR continuity of carer OR interpreter services OR language barrier* OR comorbidit* OR hypertension OR pre-eclampsia OR postpartum hemorrhage OR gestational diabetes) OR AB (sociocultural factor* OR discrimination OR racism OR systemic racism OR implicit bias OR trust OR continuity of care OR continuity of carer OR interpreter services OR language barrier* OR comorbidit* OR hypertension OR pre-eclampsia OR postpartum hemorrhage OR gestational diabetes)	• Date: 2010-2025 • Language: English • Research Articles • Humans

Screening and Data Extraction

Titles/abstracts and full texts were independently screened by two reviewers. When there were disagreements, they were arbitrated by a third reviewer.

Quality Assessment

Appropriate tools were used for the evaluation of risk of bias: Joanna Briggs Institute (JBI) checklists for cohort and cross-sectional studies [21]; Critical Appraisal Skills Programme (CASP) [22] checklist for qualitative studies; and Mixed Methods Appraisal Tool (MMAT) for mixed-methods studies [23]. The quality was reviewed independently by two reviewers and any disagreements were settled by consensus.

Data Synthesis

The approach of Popay et al. [20] was used to carry out a narrative synthesis. Given the expected variability in outcome measures, designs, and outcomes, this approach was selected. The synthesis was organized around the three pre-specified domains, namely the sociocultural domain, the clinical domain and the systemic domain. Studies were sorted into the different domains and comparisons were drawn within and across the domains.

Results

Study Selection

**Figure 1:**
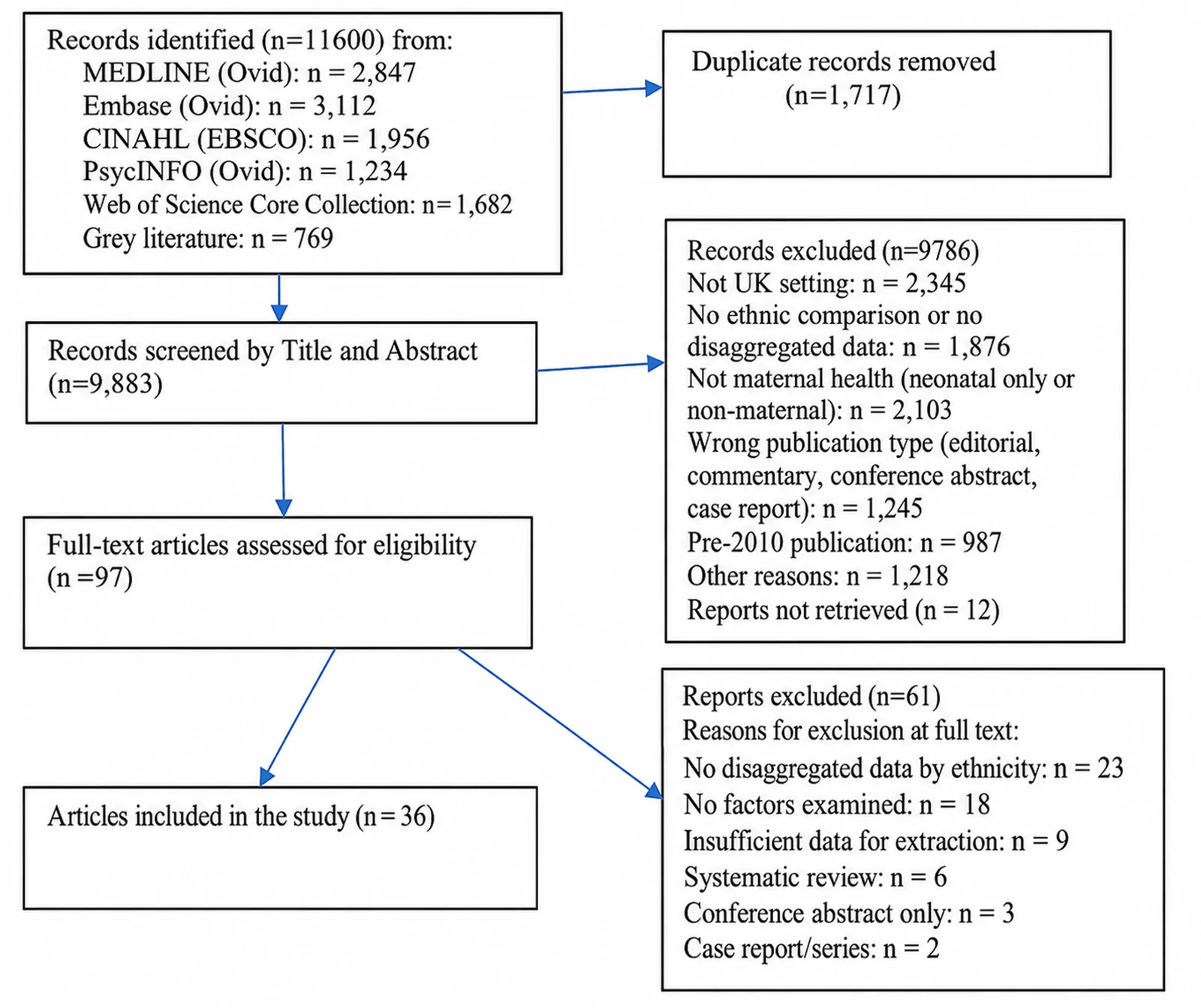
PRISMA 2020 flow diagram showing the study selection process A total of 11,600 records were identified through database searching (MEDLINE, Embase, CINAHL, PsycINFO, Web of Science) and grey literature sources. Following duplicate removal (n=1,717), 9,883 records were screened by title and abstract. Of these, 9,786 records were excluded. Full-text articles (n=97) were assessed for eligibility, resulting in the exclusion of 61 reports for reasons including no disaggregated ethnicity data (n=23), no factors examined (n=18), insufficient data (n=9), systematic reviews (n=6), conference abstracts (n=3), and case reports/series (n=2). Ultimately, 36 studies met the inclusion criteria and were included in the review. PRISMA, Preferred Reporting Items for Systematic Reviews and Meta-Analyses; MEDLINE, Medical Literature Analysis and Retrieval System Online; EMBASE, Excerpta Medica Database; CINAHL, Cumulative Index to Nursing and Allied Health Literature.

Database searches from 2010-2025 identified 11,600 records. After duplicate removal (n=1,717), 9,883 titles/abstracts were screened, with 97 full texts assessed. A total of 36 studies met inclusion criteria (Figure 1).

Study Characteristics

The 36 included studies were published between 2010 and 2025, with the majority published 2020-2025, reflecting increased research attention post-2020 MBRRACE-UK reports. Study characteristics are presented in Table 2.

**Table 2:** Characteristics of studies included in the review (2010–2025) This table summarizes the characteristics of the 36 studies included in the review of maternal health disparities among ethnic minority women in the United Kingdom. The studies were conducted across England, the wider United Kingdom, and other high-income settings between 2010 and 2025. The factors examined were categorized into clinical factors (e.g., pre-existing medical conditions, pregnancy complications, and healthcare outcomes), sociocultural factors (e.g., language barriers, cultural beliefs, health literacy, discrimination, and social support), and systemic factors (e.g., healthcare access, quality of care, institutional practices, structural inequalities, and healthcare policies). Several studies investigated multiple domains, reflecting the multifaceted nature of maternal health disparities and their determinants. UK, United Kingdom. Clinical factors refer to biological, medical, and obstetric determinants; sociocultural factors refer to social, cultural, behavioral, and communication-related determinants; systemic factors refer to healthcare system, organizational, and structural determinants.

SN	Author, Year	Setting	Factors Examined	Study Design
1	Knight et al. (2023) [1]	UK-wide	Clinical + Sociocultural + Systemic	National Report
2	Birthrights (2022) [2]	UK	Sociocultural + Systemic	Inquiry/Qualitative
3	Williams et al. (2019) [3]	UK-wide	Clinical + Sociocultural	Mixed Methods
4	Kapadia et al. (2022) [5]	UK	Systemic	Qualitative
5	MacLellan et al. (2022) [7]	UK-wide	Sociocultural + Systemic	Qualitative
6	Geddes-Barton et al. (2024) [8]	England	Clinical	Cohort
7	Cross-Sudworth et al. (2015) [9]	England	Clinical	Cohort
8	Furness et al. (2025) [10]	UK	Clinical + Sociocultural + Systemic	Qualitative
9	Docheva et al. (2023) [11]	UK	Sociocultural	Cohort
10	Cibralic et al. (2022) [12]	UK	Systemic	Cohort
11	Vousden et al. (2024) [14]	UK	Clinical	Cohort
12	Pace et al. (2022) [15]	Yorkshire, England	Systemic	Mixed Methods
13	Moreno-Santillan et al. (2025) [16]	UK	Systemic	Cohort
14	John et al. (2021) [18]	UK	Systemic	Qualitative
15	Watson et al. (2025) [24]	UK-wide	Sociocultural	Survey
16	Ajakaye (2025) [25]	England	Sociocultural	Qualitative
17	Aryasinghe et al. (2025) [26]	England	Clinical + Sociocultural	Mixed Methods
18	Conti-Ramsden et al. (2025) [27]	England	Clinical	Cohort
19	O'Shea et al. (2024) [28]	England	Clinical	Cohort
20	LoGiudice et al. (2022) [29]	UK-wide	Sociocultural + Systemic	Mixed Methods
21	Deichen Hansen et al. (2024) [30]	London, England	Sociocultural	Qualitative
22	Thomson et al. (2022) [31]	England	Clinical	Cohort
23	Liu et al. (2022) [32]	England	Clinical	Cohort
24	Jardine et al. (2022) [33]	England	Clinical	Cohort
25	Ibrahim et al. (2025) [34]	England	Clinical	Cohort
26	Middlemiss et al. (2025) [35]	England	Systemic	Cohort
27	Roebuck et al. (2025) [36]	England	Systemic	Cohort
28	Collyer and Smith (2020) [37]	England	Clinical	Mixed Methods
29	Murphy et al. (2024) [38]	UK-wide	Sociocultural + Clinical	Mixed Methods
30	Raleigh et al. (2010) [39]	England	Sociocultural	Quantitative
31	Li (2025) [40]	England	Systemic	Mixed Methods
32	Razieh et al. (2025) [41]	England	Systemic	Cohort
33	Fair et al. (2021) [42]	England	Systemic	Cross-sectional
34	Vazquez Corona et al. (2025) [43]	UK	Sociocultural	Qualitative
35	Geddes-Barton et al. (2025) [44]	England	Clinical + Sociocultural	Population-based nationwide cohort
36	Howell et al. (2018) [45]	England	Clinical	Case-Control

Quality Assessment

Quality ratings are presented in Table 3.

**Table 3:** Quality assessment of included studies This table presents the methodological quality appraisal of the 36 studies included in the review. Study quality was assessed using the Joanna Briggs Institute (JBI) critical appraisal tools, Critical Appraisal Skills Programme (CASP) checklists, and Mixed Methods Appraisal Tool (MMAT), as appropriate to the study design. Quality scores were calculated as the proportion of appraisal criteria met relative to the total number of applicable criteria and were categorized as high or moderate quality.

SN	Author, Year	Quality Assessment Tool	Criteria Met	Total Criteria	Quality Score	Rating
1	Knight et al. (2023) [1]	JBI Textual/Report	8	9	88.9%	High
2	Birthrights (2022) [2]	CASP Qualitative	9	10	90.0%	High
3	Williams et al. (2019) [3]	MMAT	18	20	90.0%	High
4	Kapadia et al. (2022) [5]	JBI CASP Qualitative	8	9	88.9%	High
5	MacLellan et al. (2022) [7]	CASP Qualitative	9	10	90.0%	High
6	Geddes-Barton et al. (2024) [8]	JBI Cohort Checklist	10	11	90.9%	High
7	Cross-Sudworth et al. (2015) [9]	JBI Cohort Checklist	7	11	63.6%	Moderate
8	Furness et al. (2025) [10]	CASP Qualitative	14	16	87.5%	High
9	Docheva et al. (2023) [11]	JBI Cohort Checklist	7	11	63.6%	Moderate
10	Cibralic et al. (2022) [12]	JBI Cohort Checklist	9	11	81.8%	High
11	Vousden et al. (2024) [14]	JBI Cohort Checklist	9	11	81.8%	High
12	Pace et al. (2022) [15]	MMAT	15	20	75.0%	Moderate
13	Moreno-Santillan et al. (2025) [16]	JBI Cohort Checklist	10	11	90.9%	High
14	John et al. (2021) [18]	CASP Qualitative	8	10	80.0%	High
15	Watson et al. (2025) [24]	CASP Qualitative	9	10	90.0%	High
16	Ajakaye (2025) [25]	CASP Qualitative	7	10	70.0%	Moderate
17	Aryasinghe et al. (2025) [26]	MMAT	18	20	90.0%	High
18	Conti-Ramsden et al. (2025) [27]	JBI Cohort Checklist	10	11	90.9%	High
19	O'Shea et al. (2024) [28]	JBI Cohort Checklist	9	11	81.8%	High
20	LoGiudice et al. (2022) [29]	MMAT	16	20	80.0%	High
21	Deichen Hansen et al. (2024) [30]	CASP Qualitative	7	10	70.0%	Moderate
22	Thomson et al. (2022) [31]	JBI Cohort Checklist	10	11	90.9%	High
23	Liu et al. (2022) [32]	JBI Cohort Checklist	10	11	90.9%	High
24	Jardine et al. (2022) [33]	JBI Cohort Checklist	14	16	87.5%	High
25	Ibrahim et al. (2025) [34]	JBI Cohort Checklist	10	11	90.9%	High
26	Middlemiss et al. (2025) [35]	JBI Cohort Checklist	9	11	81.8%	High
27	Roebuck et al. (2025) [36]	JBI Cohort Checklist	10	11	90.9%	High
28	Collyer and Smith (2020) [37]	MMAT	10	11	90.9%	High
29	Murphy et al. (2024) [38]	MMAT	16	20	80.0%	High
30	Raleigh et al. (2010) [39]	JBI Cross-sectional	8	10	80.0%	High
31	Li (2025) [40]	MMAT	14	20	70.0%	Moderate
32	Razieh et al. (2025) [41]	JBI Cohort Checklist	9	11	81.8%	High
33	Fair et al. (2021) [42]	JBI Cross-sectional	5	8	62.5%	Moderate
34	Vazquez Corona et al. (2025) [43]	CASP Qualitative	13	16	81.3%	High
35	Geddes-Barton et al. (2025) [44]	JBI Cohort Checklist	14	16	87.5%	High
36	Howell et al. (2018) [45]	JBI Case-Control	6	10	60.0%	Moderate

Of the 36 included studies, 24 (66.7%) were rated high quality and 12 (33.3%) were rated moderate quality; no studies were rated low quality. The most common methodological weaknesses included small sample sizes in qualitative studies (n=8), lack of adjustment for confounding in cohort studies (n=4), and limited generalizability from single-site studies (n=3). The predominance of high-quality ratings, particularly among cohort studies (80.0%) and national reports, strengthens confidence in the synthesized findings.

The findings of this systematic review illuminate the complex, multi-layered nature of ethnic inequalities in maternal outcomes, revealing a landscape where biological risk, interpersonal experience, and systemic design converge to produce persistently poorer outcomes for Black women. Rather than simply confirming the existence of disparities which are well-established in the literature, this discussion interprets the patterns emerging across 36 studies to understand why these disparities persist despite increased awareness, and how different forms of evidence contribute to our understanding of potential solutions.

Primacy of the Interaction: Beyond Single-Domain Explanations

Perhaps the most significant interpretation arising from this review is that sociocultural, clinical, and systemic factors do not operate independently but function as interacting elements within a complex adaptive system. The evidence consistently demonstrates that Black women navigate a care environment where biological risk factors (higher prevalence of pre-eclampsia and hemorrhage) intersect with interpersonal dynamics (dismissal of symptoms and "not being heard") and structural barriers (inconsistent service provision and the "postcode lottery"). This tripartite burden suggests that disparities cannot be adequately addressed through single-domain interventions; rather, they require coordinated action across multiple levels simultaneously.

The finding that Black women were the only group for whom continuity of carer (MCoC) models significantly reduced stillbirth rates [36] is particularly instructive. This suggests that relationship-based care may function as a protective factor specifically for populations experiencing heightened interpersonal and systemic barriers. The effect was statistically significant only for Black women, implying that continuity may buffer against the cumulative disadvantages they face, rather than being a universal improvement. However, the inconsistent implementation of MCoC across trusts [5,8,35] means that this protective mechanism is unevenly distributed, potentially paradoxically increasing inequalities between trusts with well-resourced continuity programmes and those without.

The persistence of disparities in escalation of care and timely diagnosis, even after controlling for social determinants and comorbidities [5,8,30,31], demands careful interpretation. This pattern suggests that the mechanisms driving poorer outcomes are not fully captured by measured clinical or demographic factors. The residual disparity points toward unmeasured systemic factors-implicit bias in clinical decision-making, differences in care quality, or institutional racism embedded in clinical protocols-rather than simply reflecting differences in biological risk or socioeconomic position. This interpretation is strengthened by the qualitative evidence documenting women's experiences of having their symptoms dismissed, particularly for life-threatening conditions like pre-eclampsia and postpartum hemorrhage [24].

Interpretive Power of Qualitative Evidence

The qualitative evidence in this review carries particular interpretive weight, not because it establishes causality in a statistical sense, but because it illuminates the mechanisms through which disparities are produced and experienced. The recurring theme of "not being heard" across multiple studies [6,24,25] is not merely a descriptive finding; it represents a fundamental breakdown in the therapeutic relationship that has cascading consequences for care engagement, trust, and clinical outcomes.

The pattern of symptom dismissal described by Black women, particularly regarding postpartum hemorrhage and pre-eclampsia reveals a concerning disjuncture between patients' subjective experiences and clinical responses. When women report that their concerns were ignored or that pain was not taken seriously, this suggests that clinical assessment processes may be systematically failing to incorporate patient-reported information, potentially due to implicit biases about pain tolerance, credibility, or symptom presentation among Black women. The consistent reporting of these experiences across multiple studies, time periods, and settings strengthens confidence that this represents a genuine systemic phenomenon rather than isolated instances of poor care.

The role of trust as a mediator between discrimination and care engagement is particularly significant. The evidence suggests a vicious cycle: discriminatory experiences undermine trust, reduced trust leads to delayed care-seeking or disengagement, and delayed care contributes to poorer outcomes, which may in turn reinforce negative expectations and further erode trust [11,26]. This interpretation is consistent with psychological theories of learned helplessness and mistrust in healthcare insitutions among marginalised populations. The finding that women who did not attend antenatal appointments by 24 weeks had significantly higher stillbirth rates [5] provides quantitative support for this mechanism, suggesting that disengagement from care, whether voluntary or due to barriers has measurable consequences.

Clinical Factors: Interpreting the Residual Disparity

The clinical evidence requires nuanced interpretation, particularly regarding the persistence of disparities after adjustment for measured confounders. The finding that Black women have higher rates of postpartum hemorrhage even after controlling for maternal, fetal, and birth characteristics [33], suggests that unmeasured factors - potentially including differences in care quality, timeliness of intervention, or implicit bias in clinical decision-making - contribute to outcomes beyond what would be expected based on clinical risk profiles alone.

However, the evidence for specific mechanisms is less robust. While disparities in escalation of care and timely diagnosis are well-documented [5,8,30,31], the studies do not consistently distinguish between clinician bias, differences in documentation, or systemic factors such as staffing levels or protocol design. This ambiguity means that while we can be confident that disparities exist, we have only moderate confidence in the specific mechanisms driving them. The lack of prospective studies and detailed clinical data limits our ability to parse the relative contributions of implicit bias versus structural factors versus unmeasured clinical differences.

The promising evidence for early pre-eclampsia screening using the Fetal Medicine Foundation algorithm [32] associated with a 64% decrease in stillbirth rates among South Asian women-highlights the potential of targeted, evidence-based interventions to reduce disparities. However, the inconsistent implementation of such screening across trusts underscores a critical gap between evidence and practice. This pattern suggests that disparities are not inevitable but are amenable to intervention when evidence-based protocols are consistently applied and appropriately targeted to high-risk populations.

Systemic Factors: Interpreting Implementation Gaps

The systemic evidence reveals a consistent pattern of implementation failure rather than absence of knowledge. The "postcode lottery" in service provision [26,38-40] and inconsistent collection and reporting of ethnicity data [41] suggest that the mechanisms for addressing disparities exist in policy but are not consistently operationalised in practice. This interpretation is supported by the finding that most interventions identified in previous reviews did not have a focus on race or ethnicity [5], suggesting that universal approaches may inadvertently perpetuate disparities by failing to address the specific barriers faced by minority ethnic groups.

The variation in provision of interpreter services, community outreach, and culturally tailored antenatal education [17,18] represents a structural inequity that disproportionately affects Black women. The Birthrights inquiry's characterisation of this as structural racism [6] is an important interpretive frame: rather than viewing these variations as neutral resource allocation decisions, this perspective recognises that differential access to services based on ethnicity reflects broader patterns of systemic disadvantage and institutional bias.

The evidence on continuity of carer models requires particularly careful interpretation. While MCoC was associated with reduced stillbirth rates specifically among Black women [36], the finding that implementation remains inconsistent [5,8,35] suggests that the benefits of this model are not being realised at scale. The effect may be contingent on the quality of implementation, team dynamics, and the specific resources available - factors that are not captured in routine data. This interpretation is supported by the mixed evidence on intervention effectiveness in previous reviews [41], which suggests that promising models may fail to produce consistent benefits when implemented in real-world conditions with inevitable compromises.

Significance of Temporal Context

A striking interpretation emerging from this review is the persistence of disparities despite increased awareness and policy attention since 2020. With 66.7% of included studies published in the post-2020 period, a time of heightened focus on racial inequalities following the global racial justice movements, the continued documentation of disparities suggests that awareness alone is insufficient to drive change. This pattern is consistent with broader literature on the "implementation gap" in health equity, where knowledge of problems and potential solutions does not automatically translate into improved outcomes.

The impact of the COVID-19 pandemic on maternity services [34] provides a natural experiment that illuminates the fragility of equity gains and the disproportionate impact of systemic disruptions on already marginalised populations. The finding that ethnic inequalities were exacerbated by changes to services - reduced face-to-face appointments, disruption of continuity of care, changes to screening programmes - suggests that equity requires active maintenance and cannot be assumed to be resilient to system shocks. This interpretation is strengthened by the finding that compulsory equality impact assessments were not consistently performed [34], suggesting that equity considerations are not systematically integrated into service reconfiguration decisions.

Assessing Evidence Quality: Methodological Considerations

The varying confidence levels across the three domains reflect fundamental differences in the types of evidence available rather than differences in the seriousness or importance of the problems identified. The sociocultural evidence, predominantly qualitative, is appropriately designed to explore complex experiences of discrimination and communication breakdown, providing high confidence in the existence of these phenomena and their mechanisms. However, qualitative evidence does not establish prevalence or population-level impact, creating a gap in our ability to quantify the scale of the problem or monitor progress over time.

The clinical evidence, predominantly quantitative and based on large cohort studies, provides strong evidence for the existence of disparities in outcomes but weaker evidence for their mechanisms. The reliance on retrospective cohort studies introduces concerns about residual confounding and information bias, particularly regarding ethnicity coding and clinical documentation. The clinical evidence is therefore best interpreted as establishing the magnitude of disparities that require explanation, rather than providing definitive answers about their causes.

The systemic evidence, drawing heavily on qualitative and grey literature, is strong at identifying the types of problems that exist-structural barriers, implementation gaps, inconsistent service provision, but weaker at establishing their prevalence or causal impact on outcomes. The evidence for a causal link between "postcode lottery" variation and patient outcomes is largely indirect, relying on the assumption that areas with better service provision have better outcomes. This interpretive gap highlights the need for implementation science that explicitly tests the impact of systemic changes on patient outcomes.

Strengths and Limitations

There are a number of strengths of this review. The 2010-2025 period encompasses the pre-2020 and post-2020 policy periods, and identifies if there has been evidence of change as a result of more research and policy interest. Grey literature and UK-specific literature are also included to provide good coverage of the evidence base. The triple-domain synthesis gives a comprehensive overview which would not be possible with single studies.

Limitations should be acknowledged. There was heterogeneity in study design, outcome measures, and findings, which did not allow for meta-analysis; consequently, the magnitude of effects across the domains could not be quantified. Potential publication bias exists as studies with meaningful results are more likely to be published, although some of this was addressed in our study with the inclusion of grey literature. Many of the studies included were England only, limiting generalizability to other parts of the UK (Scotland, Wales and Northern Ireland) where health systems and collections might vary. Small sample sizes were observed in qualitative studies; however, there were recurring themes found in several studies. There are few published reports, and ad hoc examples of good practice may not be published and are not disseminated throughout the maternal and neonatal health system.

Implications for Practice, Policy and Research

Clinical practice: Pain assessment and escalation pathways require attention to implicit bias and cultural competence. Trusts should implement consistent escalation strategies that account for patient communication and concerns, and all staff should be trained to identify and act on deterioration in Black women. It is necessary to develop culturally sensitive care pathways, with the participation of Black women and communities in their design.

Health policy: A national rollout of mandatory ethnicity data collection and audit should be implemented to examine disparity and hold trusts accountable. Resources should be directed toward trusts with the greatest disparities and worst implementation of continuity of carer models. National bodies must advance the workforce diversity agenda, including targets for representation of Black and minority ethnic people in senior clinical and leadership positions. The NHS Race and Health Observatory (RHO) has urged a national database to track progress on reducing poor outcomes for Black, Asian, and ethnic minority women [46]. There is a need for a specific policy on reducing ethnic health inequalities and for more funding to be invested in research on interventions that will address these inequalities. A policy direction which is welcome is outlined in the proposed SAFE Maternity Care Act which focuses on Safety, Accountability, Freedom of Choice and Equity as the core of the maternity system [47]. The Perinatal Equity and Anti-Discrimination Programme (PEADP) program, announced during UK Black Maternal Health Awareness Week (April 2026), brings together multi-disciplinary teams, offering the opportunity to enhance safety, culture and outcomes across England [48]. The program is an important step towards embedding anti-discriminatory practice in maternity services, and the observatory has produced seven anti-racism principles to make racism more visible and identify the consequences it has on people, health and outcomes.

Research: Multi-level interventions to reduce disparities must be tested by interventional studies in the UK. Longitudinal studies investigating pathways from discrimination to clinical outcomes with validated discrimination and social determinants measures are needed. Implementation science is essential to understand what will help and hinder effective implementation of continuity of carer and to ensure equity in who receives continuity. Poor evaluation and design were observed in most interventions, reflecting the need for better theoretical frameworks and clear disaggregated ethnicity classification when planning for further interventions. Reducing waste throughout the healthcare system and increasing demonstration of good practice through the development of a portal of evaluated and ongoing interventions are warranted.

## Conclusions

The disparity between the health outcomes of Black and White women in the UK represents a persistent problem that must be tackled simultaneously at sociocultural, clinical and systemic levels. Single-domain interventions have proven inadequate, as the gap has not changed over 10 years. These domains do not exist in isolation; rather, they interact, making it necessary to implement comprehensive and coordinated approaches: listening to and taking the experiences of Black women (sociocultural) seriously, ensuring that clinical care is culturally competent and equitable, and that escalation occurs when needed (clinical), and building services that offer continuity, cultural safety, and workforce diversity (systemic). 

Since 2020, the evidence base has expanded significantly, deepening understanding of the mechanisms and pathways that help sustain inequalities. However, evidence alone does not change practice. Commitment is needed from clinicians, service leaders, policymakers, and researchers to take action on this evidence. The gap will continue to exist until action is taken across all three domains at once.
